# Incarcerated lateral inguinal hernia following ilioinguinal lymph node dissection: an unusual case

**DOI:** 10.1093/jscr/rjaf107

**Published:** 2025-03-07

**Authors:** Mansha Jiwane, Elan Novis, Emily C Smartt, Thomas E Pennington

**Affiliations:** Department of Melanoma and Surgical Oncology, Royal Prince Alfred Hospital, Level 3, Gloucester House, Gloucester Drive, Camperdown, NSW 2050, Australia; Department of Melanoma and Surgical Oncology, Royal Prince Alfred Hospital, Level 3, Gloucester House, Gloucester Drive, Camperdown, NSW 2050, Australia; Department of Surgical Oncology, Melanoma Institute Australia, 40 Rocklands Road, Wollstonecraft, Sydney, NSW 2060, Australia; Division of Surgery, Faculty of Medicine and Health, The University of Sydney, NSW 2006, Australia; Department of Surgery, Northern Beaches Hospital, Frenchs Forest Rd East, Frenchs Forest, NSW 2086, Australia; Department of Melanoma and Surgical Oncology, Royal Prince Alfred Hospital, Level 3, Gloucester House, Gloucester Drive, Camperdown, NSW 2050, Australia; Department of Surgical Oncology, Melanoma Institute Australia, 40 Rocklands Road, Wollstonecraft, Sydney, NSW 2060, Australia; Division of Surgery, Faculty of Medicine and Health, The University of Sydney, NSW 2006, Australia; Department of Surgery, Northern Beaches Hospital, Frenchs Forest Rd East, Frenchs Forest, NSW 2086, Australia

**Keywords:** penile squamous cell carcinoma, lateral inguinal hernia, ilioinguinal lymph node dissection

## Abstract

Penile squamous cell carcinoma (SCC) spreads predictably from primary tumour to inguinal lymph nodes then pelvic nodes and finally, to distant sites. Inguinal dissection involves resection of all femoral and inguinal nodes and is part of the recommended management. Femoral hernias are a commonly reported consequence of these extensive dissections. This case describes an unusual hernia which developed lateral to the femoral vessels. A 68 year old man presented with penile SCC of the distal glans penis and underwent bilateral ilioinguinal node dissections for nodal recurrence. On post operative day 1, the patient developed abdominal distention and obstipation. He returned to theatre and a defect under the inguinal ligament, lateral to the femoral vessels was identified, consistent with a lateral hernia. This was repaired with a polypropylene mesh onlay. Lateral inguinal hernia is a rare occurrence after ilioinguinal node dissection but early recognition and prompt intervention can prevent significant morbidity.

## Introduction

Inguinal and pelvic lymph node dissections are part of the surgical management of penile squamous cell carcinoma (SCC) and other cutaneous malignancies including melanoma and cutaneous squamous cell carcinomas. Complications including seroma, haematoma, wound dehiscence, infection and lymphoedema are not infrequent [1, 2].

Post-operative hernias following ilio-inguinal dissection are uncommon and a hernia lateral to the femoral vessels, beneath the inguinal ligament, has not been previously reported. We report the first case following an ilio-inguinal dissection for metastatic penile SCC. This presented a diagnostic and management challenge due to proximity to the femoral neurovascular bundle, requiring a bespoke surgical repair.

We present this rare case to raise awareness of this potential complication and describe its management.

## Case report

A 68 year old man presented with a p-16 negative, moderately differentiated SCC of the distal glans penis. After 2 months, he developed recurrence with fluorodeoxyglucose (FDG)-avid left inguinal and external iliac nodes bilaterally. Clinically he had a large palpable mass in the left inguinal region and smaller palpable nodes in the right groin. After multi-disciplinary discussion, he underwent four cycles of neoadjuvant chemotherapy with no clinical or metabolic response.

He underwent bilateral ilioinguinal lymph node dissections. Left and right iliac node dissections were performed via laparoscopic, transperitoneal approach. Inguinal dissection with a sartorius transposition flap was done via a vertical groin incision. The proximal head of sartorius was mobilized to cover the femoral vessels and nerve, and was secured to the inferior edge of the inguinal ligament with a prolene suture to protect the femoral neurovascular structures in the event of wound breakdown from infection or adjuvant radiotherapy.

**Figure 1:**
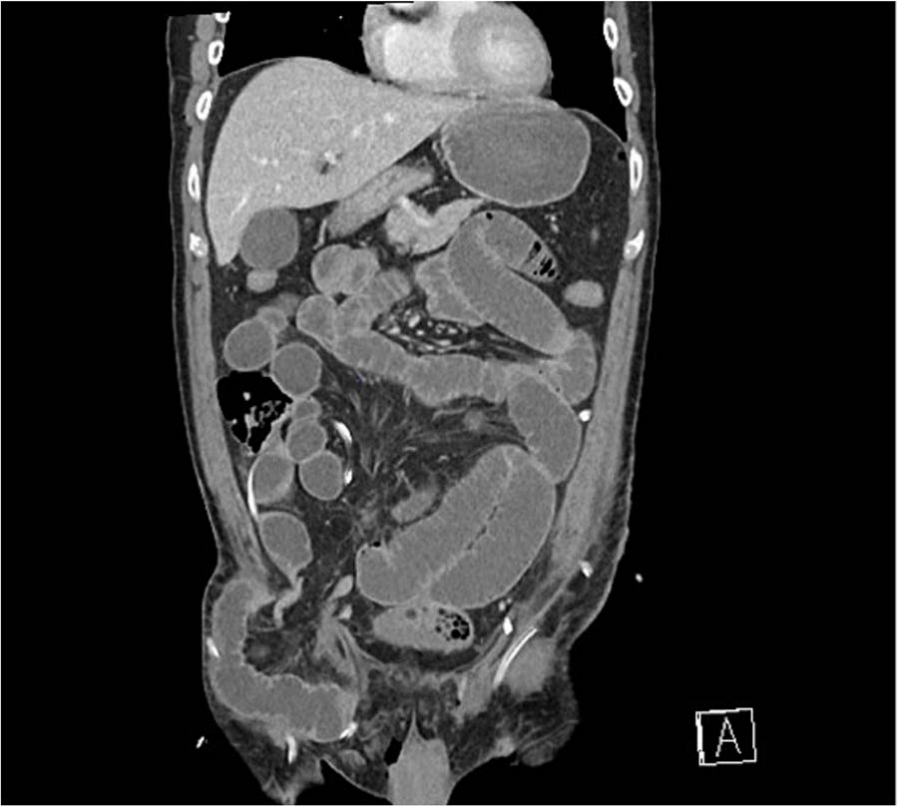
Coronal CT scan demonstrating small bowel herniating below the inguinal ligament, lateral to femoral vessels.

Progressive abdominal distension with obstipation was noted on post operative day 1. Clinical examination and an urgent CT scan of his abdomen and pelvis revealed small bowel obstruction due to herniation behind the lateral aspect of the inguinal ligament into the femoral triangle, lateral to the femoral neurovascular bundle (Fig. 1).

**Figure 2:**
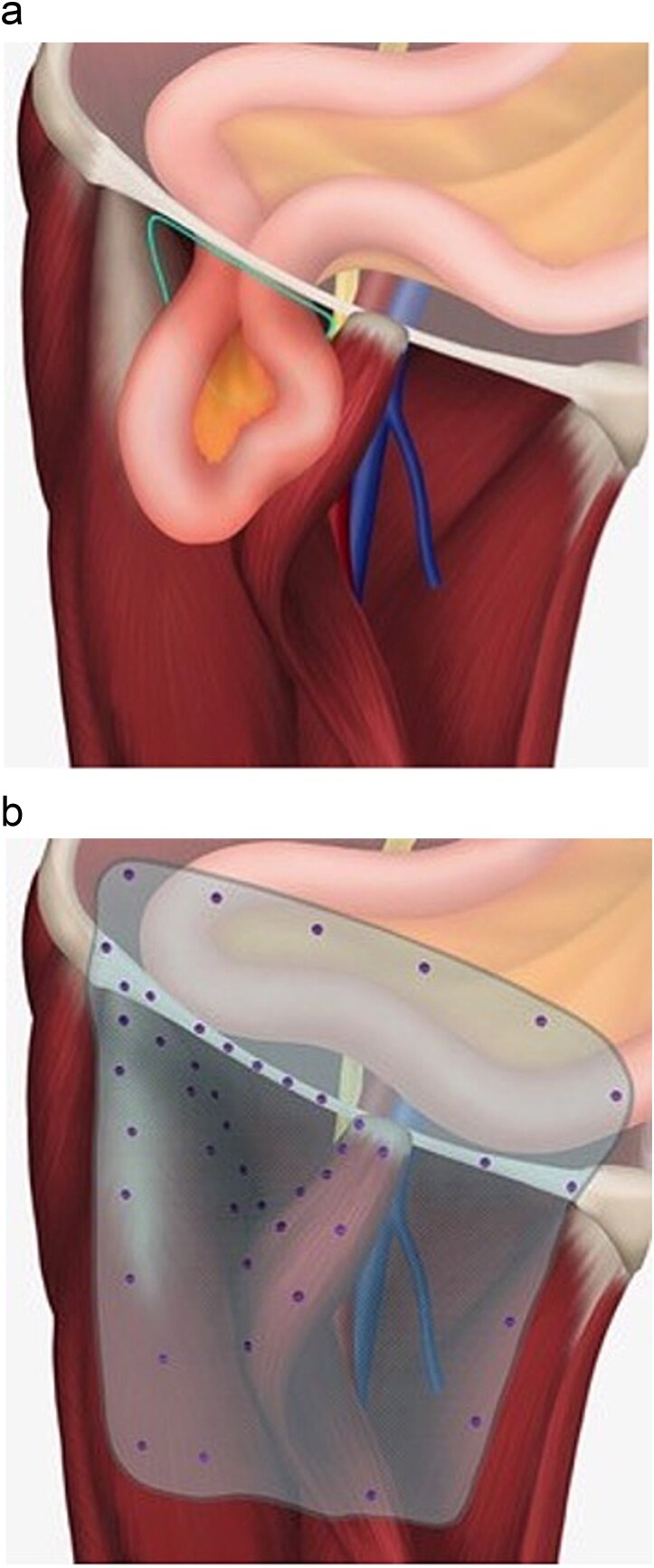
(a) Illustration demonstrating location of incarcerated hernia in relation to femoral vessels. Sartorius muscle has been transposed from anterior superior iliac spine insertion to the midpoint of the inguinal ligament to protect the femoral vessels. (b) Illustration of mesh repair and schematic placement of tacks.

The patient underwent emergency operation wherein the previous groin incision was re-opened and the herniated healthy, viable bowel reduced. An open approach was chosen due to the degree of abdominal distension, amount of herniated bowel in the femoral triangle, and concerns about difficulty in doing a preperitoneal laparoscopic mesh repair with previous iliac lymph node dissection. Intra-operatively, a large defect was identified lateral to the femoral vessels under the inguinal ligament, consistent with a lateral hernia (Fig. 2a). The sartorius flap remained intact. An onlay prolene mesh was secured to the neo-triangle created by the inguinal ligament cranially, transposed sartorius medially and rectus femoris laterally (Fig. 2b). The repair was further secured with Tisseal™ fibrin sealant. Drains were replaced and skin closed in layers.

The patient’s post-operative recovery was uneventful. At 6 months follow-up he remains free of clinical or radiological evidence of groin hernias.

## Discussion

Penile SCC accounts for more than 95% of penile cancer, which itself is an uncommon urological malignancy [3]. It spreads relatively predictably from primary tumour initially to inguinal lymph nodes, then pelvic lymph nodes and finally distant metastases [4]. The initial management of penile SCC includes treatment of the primary tumour with pathologic lymph node involvement being the most important prognostic factor. The 5 year cancer-specific survival for pN0, N1, N2, or N3 disease is 95%, 80%, 65%, and 35%, respectively [5, 6]. Ilioinguinal lymph node dissections have a complication rate of up to 80% inlcuding seroma (6%–40%), haematoma (2%–4%), wound dehiscence (17%–65%), wound infection (6%–20%), lymphoedema (22%–80%) and venous thromboembolism (5%–7%) [2, 7, 8]. The development of groin hernias following ilioinguinal lymph node dissections has rarely been reported, outside of femoral hernias [8–10]. To our knowledge this is the first reported case of a post-operative hernia developing lateral to the femoral vessels, below the inguinal ligament.

An ilioinguinal dissection combines complete resection of all femoral and inguinal lymph nodes within femoral triangle, lower 5 cms of abdominal wall superficial to the external oblique and deeper common/external iliac and obturator node groups. Ilioinguinal lymph node dissection remains the standard of care for the majority of penile SCC patients [11]. In patients with node-positive disease, only node dissection has demonstrated improved overall survival when compared to other treatment options including chemotherapy and radiotherapy [12, 13].

In this patient, a laparoscopic transperitoneal approach was adopted for the iliac/obturator nodal dissection as the senior author believes this allows for superior visualization of key structures and avoids the need for larger open incisions, particularly in patients requiring bilateral dissection. There is currently no evidence comparing laparoscopic to open technique for iliac lymph node dissection in penile SCC. It is possible that during the laparoscopic dissection, the iliopsoas fascia was disrupted, weakening the inguinal ligament attachment, permitting bowel to herniate behind it. Therefore we advocate care be taken to minimize disruption of this fascia during iliac node dissection. Extra care should be taken when dissecting between the inguinal ligament and this fascia.

This report highlights the need for awareness of lateral hernia following ilioinguinal dissection, however, the more common occurrence of femoral hernia should also be considered. These hernias are often due to failure to close the femoral canal after clearing its fibrofatty contents [10]. Extensive dissection may also compromise the anatomic integrity of the myopectineal orifice and skeletonization of the neurovascular bundles may contribute to bowel herniation and progressive enlargement of an initial defect [9, 14]. There are three reported cases in the literature of femoral hernia occurring acutely post inguinal dissection (Table 1). In each, the patient presented with abdominal pain, obstipation and vomiting between post operative days 2–6 and exploration in theatre revealed femoral hernia. Of note, all cases described have involved right sided dissection, including our case.

**Table 1:** Summary of previously published reports of postoperative hernias following inguinal dissection

**Patient**	**Operation**	**Laterality**	**Symptom onset (post-operative day)**	**Hernia relation to femoral vessels**	**Citation**
68, Male	Bilateral ilioinguinal node dissections	Right	Day 1	Lateral	Current Case
52, Female	Inguinal lymphadenectomy	Right	Day 4	Medial	Shivanna *et al.*
72, Female	Deep inguinal node dissection	Right	Day 2	Medial	Steffensen *et al.*
80, Female	Bilateral inguinal and femoral node dissection	Right	Day 6	Medial	Margina *et al.*

## Conclusions

Lateral inguinal hernia following ilio-inguinal dissection has not previously been described in the literature, but should be considered in patients who undergo both iliac/obturator nodal dissection combined with femoral/inguinal nodal dissection. Given the rarity of penile SCC and particularly, the number of patients requiring ilio-inguinal dissection, awareness of such complications is important to facilitate early diagnosis and treatment as prompt intervention can reduce significant morbidity.
